# Validation of the Neutrophil–Lymphocyte Ratio as a Mortality Risk Stratification Marker in Patients with Proximal Femoral Fractures

**DOI:** 10.3390/biomedicines14030551

**Published:** 2026-02-27

**Authors:** Alessandro Civinini, Filippo Leggieri, Marta Massenzi, Christian Carulli, Roberto Civinini, Matteo Innocenti

**Affiliations:** Orthopedic Unit, Department of Health Sciences, University of Florence, C.T.O. Largo Palagi 1, 50139 Firenze, Italy; filippoleggieri@icloud.com (F.L.); marta.massenzi@unifi.it (M.M.); christian.carulli@unifi.it (C.C.); roberto.civinini@unifi.it (R.C.); matteo.innocenti@unifi.it (M.I.)

**Keywords:** proximal femoral fractures, neutrophil-to-lymphocyte ratio, NLR, mortality, hip, arthroplasty

## Abstract

**Background/Objectives:** Proximal femoral fractures (PFF) are associated with substantial morbidity and mortality in elderly patients. Early identification of individuals at increased risk of death remains challenging. The neutrophil-to-lymphocyte ratio (NLR) is an inexpensive and readily available biomarker reflecting systemic inflammation and physiological stress, but its role as a risk stratification tool in surgically treated PFF patients is not fully established. The aim of this study was to validate NLR as a prognostic biomarker for mortality risk stratification in elderly hip fracture patients by evaluating its independent association with mortality, establishing clinically relevant risk categories, and assessing its ability to identify distinct mortality risk groups. **Methods:** This retrospective cohort study included 1113 patients aged ≥ 65 years who underwent surgery for AO/OTA 31.A (trochanteric) or 31.B (femoral neck) proximal femoral fractures between January 2021 and February 2024 at a single institution. NLR was calculated from routine admission bloodwork. The primary outcome was all-cause mortality. Kaplan–Meier survival analysis stratified patients by clinically relevant NLR categories (<5, 5–10, >10). Cox proportional hazards regression identified independent predictors of mortality. ROC analysis was performed secondarily to identify an optimal binary threshold. **Results:** At mean follow-up of 33.9 months, overall mortality was 36.2% (352/972). Stratified survival analysis demonstrated a clear dose–response relationship, with mortality rates of 26.2%, 36.5%, and 54.4% for NLR < 5, 5–10, and >10, respectively (log-rank *p* < 0.001). In multivariable Cox regression, NLR remained independently associated with mortality (HR = 1.042, 95% CI: 1.032–1.053, *p* < 0.001) after adjusting for age and time to surgery. ROC analysis identified an optimal binary cut-off of 6.59 (AUC 0.614). **Conclusions:** Elevated preoperative NLR is independently associated with increased mortality following surgery for proximal femoral fractures, particularly in very elderly patients. Given its simplicity and universal availability, NLR may represent a useful adjunct for early perioperative risk stratification.

## 1. Introduction

Proximal femoral fracture (PFF) is a common and devastating injury with substantial implications for global public health economies, particularly with the aging of populations worldwide. In the United States, the economic burden of hip fractures is amongst the top 20 expensive diagnoses, with approximately 20 billion dollars spent annually on the management of this injury [1,2,3]. It is estimated that there will be approximately 300,000 hip fracture cases annually in the United States by the year 2030 [4]. Despite early surgery time protocols (≤36 h after injury), PFF still represents a major life-threatening event, especially in the elderly, with a mortality rate of 10% within the first 30 days of admission and 30% at 1 year [5,6]; furthermore, PFF ranks among the top 10 causes of disability in the elderly population [7]. Thus, rapid clinical tools that enable quantitative and individualized risk assessment are needed to help orthopedic surgeons tailor and optimize patient management.

Several independent factors have been shown to influence short-term survival following a hip fracture. Baseline comorbidities, pre-fracture cognitive function, baseline mobility, living situation, male sex, and increasing age are all associated with elevated mortality risk [8]. Likewise, laboratory biomarkers such as hemoglobin, lymphocyte count, and serum albumin have been recognized as significant predictors of early postoperative mortality in hip fracture patients. Moreover, decreased albumin and lymphocyte levels are also linked to worse survival outcomes in other settings, including chronic kidney disease requiring dialysis, cancer, and even among elderly individuals without underlying medical conditions [9]. Hypoalbuminemia prior to surgery has additionally been associated with delayed recovery, reduced postoperative ambulation, and a higher incidence of complications [10,11,12].

The neutrophil–lymphocyte ratio (NLR) is a inexpensive, simple, rapidly responsive and easily available parameter of body stress response and inflammation status [13,14], yet its application in orthopedic surgery has been investigated primarily as a prognostic marker for prosthetic joint infections [15,16,17], with limited exploration of its role in predicting specific postoperative outcomes such as mortality and complications following PFF surgery.

An elevated neutrophil-to-lymphocyte ratio has been consistently associated with unfavorable outcomes and increased mortality across a broad spectrum of clinical settings. In orthopedics, higher NLR values have been linked to worse prognostic outcomes in various musculoskeletal conditions [17,18]. Beyond orthopedic practice, the prognostic relevance of NLR has been extensively documented in several non-orthopedic fields, including cardiovascular diseases [19,20,21,22,23,24], renal disorders [25,26], chronic obstructive pulmonary disease [27], inflammatory conditions [28,29], oncologic diseases [30,31,32,33], and diverse postoperative settings [34]. Rather than representing a disease-specific biomarker, NLR may reflect an underlying state of biological vulnerability, immune dysregulation, and frailty, particularly in elderly patients exposed to acute surgical stress. In this context, NLR should be interpreted as a risk stratification marker rather than a standalone predictive test.

We therefore hypothesized that preoperative NLR could serve as a simple, accessible prognostic biomarker for mortality risk stratification in elderly patients undergoing surgery for PFF. The aim of this study was to validate NLR as a risk stratification tool by (1) evaluating its association with all-cause mortality after adjusting for key demographic factors, (2) establishing clinically relevant risk categories based on NLR levels, and (3) assessing its ability to identify distinct mortality risk groups in a real-world surgical population.

## 2. Materials and Methods

### 2.1. Design and Population

We retrospectively reviewed data from a population of patients who underwent surgery for a proximal femoral fracture between January 2021 and February 2024 at a single Institution. The study was conducted in accordance with the Declaration of Helsinki. The study protocol was formally reviewed by the Institutional Review Board, which granted a waiver of the requirement for ethics approval and informed consent (IRB reference number: CEAVC22307). Inclusion criteria were: age ≥ 65 years, radiologically confirmed diagnosis of proximal femoral fracture (AO/OTA 31.A, intertrochanteric fractures, and 31.B, femoral neck fractures), complete baseline laboratory data, and minimum potential follow-up of 12 months from date of surgery. Patients who died before 12 months were included as outcome events in the survival analysis, while patients who were alive but lost to follow-up before 12 months without a recorded death were excluded.

The final study cohort included 1113 patients, including 763 women (68.6%) and 350 men (31.4%). There were 623 patients (56.0%) with AO/OTA 31.A type fractures and 490 (44.0%) with AO/OTA 31.B. Overall, 706 patients (63.4%) were treated with internal fixation, while the remaining 407 patients (36.6%) underwent hip arthroplasty procedures.

### 2.2. Data Collection

Completely anonymized data were extracted from electronic medical records. Demographic variables included age at the time of the intervention, diagnosis, date of admission, date of surgery, date of discharge, date of death, neutrophil count, and leucocyte count. Time to surgery was calculated as the interval between admission and the start of the surgical procedure, measured in days. NLR was calculated by dividing the absolute neutrophil count by the absolute lymphocyte count from the same blood sample. All blood samples were collected at admission and processed at the same Institution’s General Laboratory Department.

### 2.3. Outcome Measures

This study validated NLR as a prognostic biomarker for mortality risk stratification to assess whether NLR remained independently associated with all-cause mortality after adjusting for age and time to surgery, define clinically relevant NLR categories based on biological rationale and cohort distribution to stratify patients into distinct risk groups, and evaluate whether these NLR categories identified groups with clinically meaningful differences in mortality rates and survival times.

### 2.4. Statistical Analysis

Statistical analysis was performed using SPSS software 31.0.0. *p*-value < 0.05 was considered statistically significant. Variables were reported using means, standard deviations, medians, ranges, and interquartile ranges (Q1–Q3). Categorical variables were summarized as frequencies and percentages. Kaplan–Meier survival curves were stratified using clinically relevant NLR categories (0–5, 5–10, >10) to assess risk stratification capability. These thresholds were selected based on established biological rationale from the literature [35,36]. Cumulative survival probabilities were estimated at 12, 24, 36, and 48 months. Survival differences between groups were assessed using the log-rank test. ROC curve analysis was performed to identify an optimal binary threshold and facilitate comparison with the existing literature. The area under the curve (AUC) with 95% confidence interval was calculated. We used Youden’s index to determine the optimal NLR cut-off value, for which we calculated sensitivity, specificity, positive predictive value (PPV), and negative predictive value (NPV). Cox proportional hazards regression was used to identify independent predictors of mortality, adjusting for age and time to surgery. Hazard ratios (HR) with 95% confidence intervals were calculated. The proportional hazards assumption was tested using Schoenfeld residuals and was satisfied (global test *p* = 0.84). Patients who were lost to follow-up and those who died before the final follow-up without implant failure were included as censored data in the survival analysis.

## 3. Results

Baseline characteristics are provided in Table 1. The mortality rate at the final follow up was 35.8% (N = 399). The mean survival time was 33.8 months (SE = 0.6, 95% CI: 32.6–34.3) (Table 2).

After adjusting for age and time to surgery in Cox proportional hazards regression, NLR remained independently associated with mortality (HR = 1.04, 95% CI: 1.03–1.05, *p* < 0.001) (Table 3). Each unit increase in NLR was associated with a 4.2% increase in the hazard of death. Age was also a significant independent predictor (HR = 1.06, 95% CI: 1.04–1.07, *p* < 0.001), while time to surgery demonstrated a significant association (HR = 1.06, 95% CI: 1.03–1.09, *p* < 0.001).

**Figure 1:**
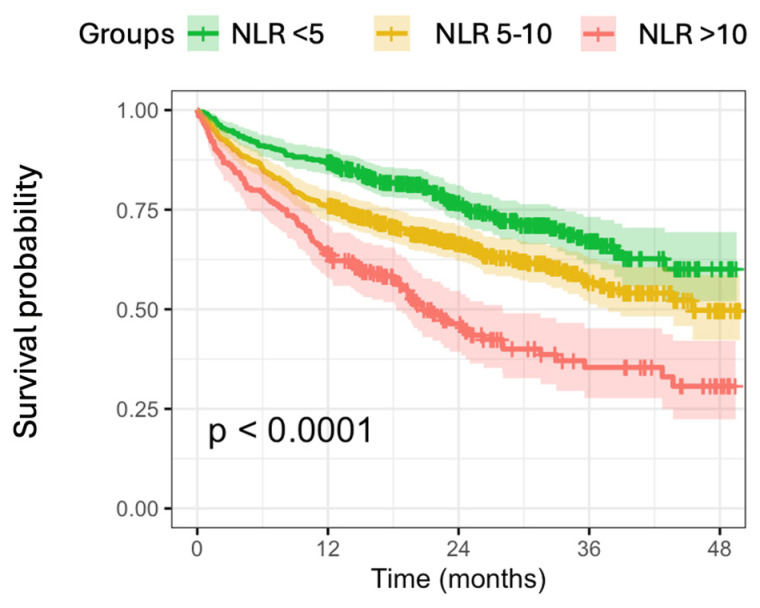
Kaplan–Meier survival curves stratified by neutrophil-to-lymphocyte ratio (NLR) categories. Shaded areas represent 95% confidence intervals. The risk table below shows the number of patients at risk at each time point.

Kaplan–Meier survival analysis stratified by clinically relevant NLR categories demonstrated a clear dose–response relationship between NLR levels and mortality risk (Figure 1). Patients were distributed as follows: NLR < 5 (n = 424, 38.1%), NLR 5–10 (n = 485, 43.6%), and NLR > 10 (n = 204, 18.3%). Mortality rates increased progressively across strata: 26.2% in the NLR < 5 group, 36.5% in the NLR 5–10 group, and 54.4% in the NLR > 10 group (log-rank test: χ^2^ = 56.8, *p* < 0.001). Median survival was not reached in the lowest NLR group, while it was 45.5 months (95% CI: 37.7-NA) in the intermediate group and 20.7 months (95% CI: 18.3–28.1) in the highest NLR group, reflecting the substantial prognostic gradient across categories.

The stratified analysis of NLR hazard ratio by age group revealed varying associations across different age brackets. While no significant associations were found in patients aged 60–69 years (HR = 1.2, *p* = 0.139) and 70–79 years (HR = 0.9, *p* = 0.793,), significant associations emerged in older populations. Among octogenarians (HR = 1.03, *p* < 0.001) and nonagenarians (HR = 1.05, *p* < 0.001), NLR demonstrated strong prognostic value, while data for centenarians were insufficient for hazard ratio calculation.

**Figure 2:**
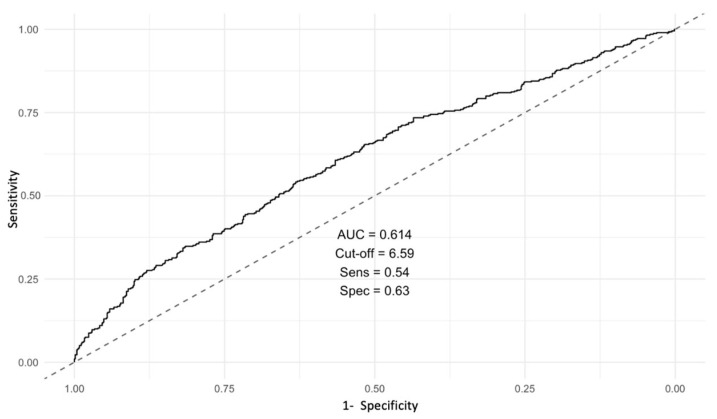
ROC curve showing the discriminative ability of admission neutrophil-to-lymphocyte ratio (NLR) for all-cause mortality.

ROC curve analysis was performed, showing the optimal cut-off at 6.59 (AUC 0.614, 95% CI: 0.579–0.649) (Figure 2), with a sensitivity of 60.7% and specificity of 56.3% (Table 4).

## 4. Discussion

This study validated NLR as a prognostic biomarker for mortality risk stratification in elderly patients undergoing surgery for PFF. Our findings demonstrate that NLR successfully identifies clinically distinct mortality risk groups, with mortality rates ranging from 26.2% (NLR < 5) to 54.4% (NLR > 10), representing more than a two-fold risk difference with substantial survival separation. This was a biomarker validation study focused on risk stratification, categorizing patients into clinically meaningful risk groups to inform care decisions.

NLR has already been validated as a prognostic and diagnostic tool across several medical subspecialties [37]; Song et al. demonstrated a significant association between elevated NLR and increased all-cause mortality in a representative sample of the U.S. population, highlighting its relevance as a general marker of systemic risk [14].

In orthopedics, prior investigations have primarily focused on the role of NLR in predicting periprosthetic joint infections [15,16]. However, its application as a broader prognostic tool in PFF surgery remains limited, with Morom et al. reporting an association between elevated NLR and mortality in PFF patients [38].

ROC curve analysis identified an optimal binary cut-off of 6.59 in the current study. This threshold is consistent with previously reported values in hip fracture populations. This threshold is consistent with previously reported values in the hip fracture literature, where NLR cut-offs typically range from 5 to 8.5, reflecting context- and population-specific variations. Fisher et al. demonstrated that an NLR greater than 5 independently predicted postoperative myocardial injury in 415 orthogeriatric patients, while values exceeding 8.5 were associated with an increased risk of infection and in-hospital mortality [39]. Ozbek et al. found that, in patients treated with intramedullary nailing for PFF, an NLR threshold greater than 5.25 was significantly correlated with higher mortality at one-year follow-up [40]. Our cut-off of 6.59 falls within this established range, though we emphasize that optimal thresholds may vary depending on population characteristics, comorbidity burden, and institutional practices. For this reason, we advocate for categorical risk stratification (NLR < 5, 5–10, >10) based on biological rationale rather than reliance on a single binary threshold, as this approach better captures the dose–response relationship between NLR and mortality observed in our cohort.

Previous smaller cohort studies have also demonstrated prognostic associations. Alexandru et al. evaluated 148 patients with diaphyseal fractures, reporting significantly higher NLR values in individuals with femoral fractures and a positive association with length of hospital stay [41]. Temiz et al. reported that elevated NLR was significantly associated with increased one-year mortality among 50 female patients with PFF [42].

This association between elevated NLR and increased mortality may be attributed to the role of NLR as a surrogate marker of the body’s overall inflammatory and physiological stress response [43]. An elevated neutrophil count reflects an upregulated innate immune response and heightened systemic inflammation [44], while a reduced lymphocyte count may indicate impaired adaptive immunity and stress-induced immunosuppression [45]. This immune imbalance compromises recovery from surgical trauma and resistance to postoperative complications, particularly in elderly frail patients where dysregulated inflammation exacerbates pre-existing comorbidities.

The age-dependent prognostic value of NLR, with significant associations only in octogenarians and nonagenarians, likely reflects interconnected mechanisms characteristic of advanced age. ‘Inflammaging,’ the chronic low-grade inflammatory state of advanced age, amplifies acute inflammatory markers through persistent innate immune activation and declining adaptive immunity. Very elderly patients have reduced physiological reserves and increased frailty burden, making them vulnerable to the acute-on-chronic inflammatory challenge imposed by fracture and surgical trauma. Age-related immune dysregulation compromises adaptive immunity while maintaining innate activation, manifesting as elevated NLR with diminished stress response capacity.

Systemic inflammation links to adverse biological phenotypes and frailty. Ozturk et al. demonstrated, in 1635 elderly individuals, that elevated NLR was independently associated with reduced bone mineral density and served as a predictor of osteoporosis [46]. Also, chronic low-grade inflammation plays a central role in bone remodeling and the pathophysiology of osteoporosis [47,48], as well as in the development of atherosclerosis [49]. Although NLR has gained increasing attention as a prognostic biomarker across various medical disciplines, the literature still lacks a universally accepted threshold that distinguishes physiological from pathological levels. Forget et al. established reference values for NLR in a healthy adult population, identifying a normal range between 0.78 and 3.53, based on blood samples from over 400 individuals without known comorbidities or systemic inflammation [50]

The overall mortality rate in our cohort was 35.8%, consistent with literature reporting 30% one-year mortality in elderly PFF patients [5,6]. Importantly, our stratified analysis by age group revealed that NLR was not a significant predictor of mortality in patients aged below 80. This suggests that the prognostic relevance of NLR may be age-dependent, likely due to the interaction between systemic inflammatory response and underlying frailty, immune dysfunction, or comorbidity burden in older patients.

NLR enables immediate risk stratification from routine admission bloodwork without requiring a comprehensive geriatric assessment, which is often unavailable in emergency settings [51]. Patients with NLR > 10 and age ≥ 80 face a 54% mortality risk, warranting enhanced monitoring, early geriatric co-management, and proactive care planning. NLR does not replace clinical judgment—it provides an objective, quantitative signal to identify who needs heightened attention, enabling risk-stratified care from admission onward rather than reactive management of deterioration. The clinical utility of NLR lies in its simplicity and cost-effectiveness. Unlike more complex scoring systems, NLR can be calculated quickly from routine blood tests performed on admission

This study should acknowledge some limitations. The retrospective single-center design introduces potential selection and information biases, and external validation in diverse healthcare systems is needed before widespread clinical implementation. Our database lacked comprehensive data on frailty scores, detailed comorbidity indices, nutritional parameters, cognitive function, and pre-fracture functional status. We did not systematically record potential acute confounders of NLR, such as active infection at admission, recent corticosteroid use, or acute inflammatory conditions, which may transiently elevate NLR independent of underlying frailty. However, for risk stratification biomarker validation, the fact that NLR successfully stratified risk in heterogeneous real-world patients despite these unmeasured variables demonstrates robust generalizability in routine clinical conditions. We did not systematically collect cause-specific mortality or detailed complication data, though all-cause mortality remains the most clinically relevant endpoint for risk stratification in elderly hip fracture patients, where deaths typically result from multifactorial causes.

Future prospective, multicenter studies with comprehensive covariate collection are needed to validate these risk categories externally and clarify NLR’s incremental value beyond multidomain geriatric assessment. Also, future studies should integrate NLR with validated frailty indices to determine whether NLR provides incremental prognostic value beyond multidomain frailty assessment.

## 5. Conclusions

An elevated neutrophil-to-lymphocyte ratio at hospital admission is a reliable and independent prognostic biomarker for mortality risk stratification in octogenarian and nonagenarian patients undergoing surgery for proximal femoral fractures.

Categorical stratification using NLR thresholds of 5 and 10 successfully identified clinically distinct mortality risk groups with significantly higher mortality rates during follow-up, reflecting the impact of systemic inflammatory stress in this vulnerable population.

Given its simplicity, low cost, and availability from routine blood tests, NLR represents a valuable adjunct to existing clinical risk assessment tools. Its integration into preoperative evaluation protocols may aid in early identification of high-risk patients, thereby facilitating individualized perioperative management strategies aimed at reducing complications and improving survival outcomes.

## Figures and Tables

**Table 1:** Baseline characteristics and differences between deaths and survivals.

	Survivals (N = 714)							Deaths (n = 399)							
	Mean	SD	Min	Max	Q1	Median	Q3	Mean	SD	Min	Max	Q1	Median	Q3	*p*
Age (years)	82.2	10.2	38.2	101.5	77.0	84.3	89.5	87.1	8.5	44.0	107.0	82.7	88.7	92.9	<0.001
Neu (10^9^/L)	7.0	2.9	0.7	28.8	5.1	6.4	8.4	7.9	3.6	1.2	26.5	5.4	7.2	9.7	<0.001
Lym (10^9^/L)	1.3	1.6	0.3	7.45	0.9	1.2	1.6	1.2	2.2	0.1	31.3	0.7	1.0	1.3	<0.001
NLR	6.6	4.3	0.2	45.5	3.9	5.6	8.0	9.3	9.5	0.3	107.6	4.7	6.9	10.5	<0.001
FU (Months)	26.7	10.3	12.0	49.8	17.8	24.7	33.8	11.7	10.3	0.0	45.5	3.3	8.7	18.1	<0.001

*p*-values from Mann–Whitney U test for continuous variables and chi-square test for categorical variables. NLR = neutrophil-to-lymphocyte ratio; Neu = neutrophils; Lym = lymphocytes; FU = follow-up.

**Table 2:** Survival and mortality rates at specific time points (%).

Time (Months)	Survival Probability	Mortality Rate	Standard Error	Lower 95% CI	Upper 95% CI	Number at Risk	Cumulative Events
12	78.0	22.0	1.2	75.6	78.0	868	245
24	66.8	33.2	1.5	63.9	66.8	423	343
36	56.9	43.1	1.9	53.2	56.9	164	388
48	50.0	50.0	2.7	45.0	50.0	22	399

**Table 3:** Multivariate Cox proportional hazards regression model assessing the impact of NLR on mortality.

Variable	Estimate	SE	Z value	*p*	HR	95% CI
**Age**	0.06	0.007	8.3	<0.001	1.06	1.04–1.07
**NLR**	0.04	0.005	9.0	<0.001	1.04	1.03–1.05
**Time to Surgery**	0.001	0	1.9	0.06	1.0	1.0–1.001

HR = hazard ratio; CI = confidence interval.

**Table 4:** Performance at different NLR cut-offs.

NLR Cut-Off	Sensitivity	Specificity	PPV	NPV	Accuracy
3	93.5	11.8	37.2	76.4	41.1
4	81.7	25.9	38.1	71.7	45.9
5	71.9	43.8	41.7	73.6	53.9
6	60.7	56.3	43.7	71.9	57.9
7	49.6	66.5	45.3	70.3	60.5
8	39.3	75.4	47.1	69.0	62.4
9	34.1	81.9	51.3	69.0	64.8
10	27.6	87.1	54.5	68.3	65.8

## Data Availability

The data presented in this study are available on reasonable request from the corresponding author. The data are not publicly available due to privacy and ethical restrictions.

## Abbreviations

PFF	Proximal femoral fracture
NLR	Neutrophil-to-lymphocyte ratio
ROC	Receiver operating characteristic
AUC	Area under the curve
OR	Odds ratio
HR	Hazard ratio
CI	Confidence interval
PPV	Positive predictive value
NPV	Negative predictive value
FU	Follow-up
FUm	Follow-up in months
ORIF	Open reduction and internal fixation

## References

[1] Brauer C.A., Coca-Perraillon M., Cutler D.M., Rosen A.B. (2009). Incidence and mortality of hip fractures in the United States. JAMA.

[2] Shimizu T., Miyamoto K., Masuda K., Miyata Y., Hori H., Shimizu K., Maeda M. (2007). The clinical significance of impaction at the femoral neck fracture site in the elderly. Arch. Orthop. Trauma Surg..

[3] Miyamoto R.G., Kaplan K.M., Levine B.R., Egol K.A., Zuckerman J.D. (2008). Surgical management of hip fractures: An evidence-based review of the literature. I: Femoral neck fractures. J. Am. Acad. Orthop. Surg..

[4] Brox W.T., Roberts K.C., Taksali S., Wright D.G., Wixted J.J., Tubb C.C., Patt J.C., Templeton K.J., Dickman E., Adler R.A. (2015). The American Academy of Orthopaedic Surgeons Evidence-Based Guideline on Management of Hip Fractures in the Elderly. J. Bone Jt. Surg..

[5] Giummarra M.J., Ekegren C.L., Gong J., Simpson P., Cameron P.A., Edwards E., Gabbe B.J. (2020). Twelve month mortality rates and independent living in people aged 65 years or older after isolated hip fracture: A prospective registry-based study. Injury.

[6] Neuburger J., Currie C., Wakeman R., Tsang C., Plant F., De Stavola B., Cromwell D.A., van der Meulen J. (2015). The Impact of a National Clinician-led Audit Initiative on Care and Mortality after Hip Fracture in England: An External Evaluation using Time Trends in Non-audit Data. Med. Care.

[7] Bhandari M., Swiontkowski M. (2017). Management of Acute Hip Fracture. N. Engl. J. Med..

[8] Stewart N.A., Chantrey J., Blankley S.J., Boulton C., Moran C.G. (2011). Predictors of 5 year survival following hip fracture. Injury.

[9] Bajada S., Smith A., Morgan D. (2015). Pre-operative nutritional serum parameters as predictors of failure after internal fixation in undisplaced intracapsular proximal femur fractures. Injury.

[10] Sim S.D., Sim Y.E., Tay K., Howe T.S., Png M.A., Chang C.C.P., Abdullah H.R., Koh J.S.B. (2021). Preoperative hypoalbuminemia: Poor functional outcomes and quality of life after hip fracture surgery. Bone.

[11] Bohl D.D., Shen M.R., Hannon C.P., Fillingham Y.A., Darrith B., Valle C.J.D. (2017). Serum albumin predicts survival and postoperative course following surgery for geriatric hip fracture. J. Bone Jt. Surg..

[12] Aldebeyan S., Nooh A., Aoude A., Weber M.H., Harvey E.J. (2017). Hypoalbuminaemia—A marker of malnutrition and predictor of postoperative complications and mortality after hip fractures. Injury.

[13] Buonacera A., Stancanelli B., Colaci M., Malatino L. (2022). Neutrophil to Lymphocyte Ratio: An Emerging Marker of the Relationships between the Immune System and Diseases. Int. J. Mol. Sci..

[14] Song M., Graubard B.I., Rabkin C.S., Engels E.A. (2021). Neutrophil-to-lymphocyte ratio and mortality in the United States general population. Sci. Rep..

[15] Salimi M., Karam J.A., Willman M., Willman J., Lucke-Wold B., Khanzadeh S., Mirghaderi P., Parvizi J. (2024). Neutrophil to Lymphocyte Ratio and Periprosthetic Joint Infection: A Systematic Review and Meta-Analysis. J. Arthroplast..

[16] Nairn L., Sivaratnam S., Bali K., Wood T.J. (2024). Neutrophil to Lymphocyte Ratio as an Indicator of Periprosthetic Joint Infection: A Retrospective Cohort Study. J. Am. Acad. Orthop. Surg..

[17] Sigmund I.K., Puchner S.E., Windhager R. (2021). Serum Inflammatory Biomarkers in the Diagnosis of Periprosthetic Joint Infections. Biomedicines.

[18] Kaymaz B., Büyükdogan K., Kaymaz N., Kömürcü E., Golge U.H., Goksel F., Aksoy M.C. (2016). Neutrophil to lymphocyte ratio may be a predictive marker of poor prognosis in Legg-Calvé-Perthes disease. HIP Int..

[19] Wang X., Fan X., Ji S., Ma A., Wang T. (2018). Prognostic value of neutrophil to lymphocyte ratio in heart failure patients. Clin. Chim. Acta.

[20] Shao Q., Chen K., Rha S.W., Lim H.E., Li G., Liu T. (2015). Usefulness of Neutrophil/Lymphocyte Ratio as a Predictor of Atrial Fibrillation: A Meta-analysis. Arch. Med. Res..

[21] Tamhane U.U., Aneja S., Montgomery D., Rogers E.K., Eagle K.A., Gurm H.S. (2008). Association Between Admission Neutrophil to Lymphocyte Ratio and Outcomes in Patients With Acute Coronary Syndrome. Am. J. Cardiol..

[22] Balta S., Celik T., Mikhailidis D.P., Ozturk C., Demirkol S., Aparci M., Iyisoy A. (2016). The Relation Between Atherosclerosis and the Neutrophil-Lymphocyte Ratio. Clin. Appl. Thromb./Hemost..

[23] Bhat T.M., Afari M.E., Garcia L.A. (2016). Neutrophil lymphocyte ratio in peripheral vascular disease: A review. Expert Rev. Cardiovasc. Ther..

[24] Niculescu R., Russu E., Arbănași E.M., Kaller R., Arbănași E.M., Melinte R.M., Coșarcă C.M., Cocuz I.G., Sabău A.H., Tinca A.C. (2022). Carotid Plaque Features and Inflammatory Biomarkers as Predictors of Restenosis and Mortality Following Carotid Endarterectomy. Int. J. Environ. Res. Public Health.

[25] Woziwodzka K., Dziewierz A., Pawica M., Panek A., Krzanowski M., Gołasa P., Latacz P., Burkat M., Kuźniewski M., Krzanowska K. (2019). Neutrophil-to-lymphocyte ratio predicts long-term all-cause mortality in patients with chronic kidney disease stage 5. Folia Med. Crac..

[26] Mureșan A.V., Russu E., Arbănași E.M., Kaller R., Hosu I., Arbănași E.M., Voidăzan S.T. (2022). The Predictive Value of NLR, MLR, and PLR in the Outcome of End-Stage Kidney Disease Patients. Biomedicines.

[27] Paliogiannis P., Fois A.G., Sotgia S., Mangoni A.A., Zinellu E., Pirina P., Negri S., Carru C., Zinellu A. (2018). Neutrophil to lymphocyte ratio and clinical outcomes in COPD: Recent evidence and future perspectives. Eur. Respir. Rev..

[28] Chandrashekara S., Mukhtar Ahmad M., Renuka P., Anupama K.R., Renuka K. (2017). Characterization of neutrophil-to-lymphocyte ratio as a measure of inflammation in rheumatoid arthritis. Int. J. Rheum. Dis..

[29] Kim D.S., Shin D., Lee M.S., Kim H.J., Kim D.Y., Kim S.M., Lee M.G. (2016). Assessments of neutrophil to lymphocyte ratio and platelet to lymphocyte ratio in Korean patients with psoriasis vulgaris and psoriatic arthritis. J. Dermatol..

[30] Guthrie G.J.K., Charles K.A., Roxburgh C.S.D., Horgan P.G., McMillan D.C., Clarke S.J. (2013). The systemic inflammation-based neutrophil-lymphocyte ratio: Experience in patients with cancer. Crit. Rev. Oncol. Hematol..

[31] Miyamoto R., Inagawa S., Sano N., Tadano S., Adachi S., Yamamoto M. (2018). The neutrophil-to-lymphocyte ratio (NLR) predicts short-term and long-term outcomes in gastric cancer patients. Eur. J. Surg. Oncol..

[32] Yang J.J., Hu Z.G., Shi W.X., Deng T., He S.Q., Yuan S.G. (2015). Prognostic significance of neutrophil to lymphocyte ratio in pancreatic cancer: A meta-analysis. World J. Gastroenterol..

[33] Bojaxhiu B., Templeton A.J., Elicin O., Shelan M., Zaugg K., Walser M., Giger R., Aebersold D.M., Dal Pra A. (2018). Relation of baseline neutrophil-to-lymphocyte ratio to survival and toxicity in head and neck cancer patients treated with (chemo-) radiation. Radiat. Oncol..

[34] Vaughan-Shaw P.G., Rees J.R.E., King A.T. (2012). Neutrophil lymphocyte ratio in outcome prediction after emergency abdominal surgery in the elderly. Int. J. Surg..

[35] Gu X.B., Tian T., Tian X.J., Zhang X.J. (2015). Prognostic significance of neutrophil-to-lymphocyte ratio in non-small cell lung cancer: A meta-analysis. Sci. Rep..

[36] Dong C.H., Wang Z.M., Chen S.Y. (2018). Neutrophil to lymphocyte ratio predict mortality and major adverse cardiac events in acute coronary syndrome: A systematic review and meta-analysis. Clin. Biochem..

[37] Lowsby R., Gomes C., Jarman I., Lisboa P., Nee P.A., Vardhan M., Eckersley T., Saleh R., Mills H. (2015). Neutrophil to lymphocyte count ratio as an early indicator of blood stream infection in the emergency department. Emerg. Med. J..

[38] Marom O., Paz I., Segal D., Topaz G., Abelson N., Tavdi A., Behrbalk R., Palmanovich E., Ohana N., Yaacobi E. (2023). Proximal Femur Fractures in the Elderly—A Novel Modality to Predict Mortality: The Neutrophil-to-Lymphocyte Ratio. J. Clin. Med..

[39] Fisher A., Srikusalanukul W., Fisher L., Smith P. (2016). The Neutrophil to Lymphocyte Ratio on Admission and Short-Term Outcomes in Orthogeriatric Patients. Int. J. Med. Sci..

[40] Özbek E.A., Ayanoğlu T., Olçar A., Yalvaç S. (2020). Is the preoperative neutrophil-to-lymphocyte ratio a predictive value for postoperative mortality in orthogeriatric patients who underwent proximal femoral nail surgery for pertrochanteric fractures?. Ulus. Travma Acil Cerrahi Derg..

[41] Alexandru L., Haragus H., Deleanu B., Timar B., Poenaru D.V., Vlad D.C. (2019). Haematology panel biomarkers for humeral, femoral, and tibial diaphyseal fractures. Int. Orthop..

[42] Temiz A., Ersözlü S. (2019). Admission neutrophil-to-lymphocyte ratio and postoperative mortality in elderly patients with hip fracture. Ulus. Travma Acil Cerrahi Derg..

[43] Mortaz E., Alipoor S.D., Adcock I.M., Mumby S., Koenderman L. (2018). Update on neutrophil function in severe inflammation. Front. Immunol..

[44] Li Y., Wang W., Yang F., Xu Y., Feng C., Zhao Y. (2019). The regulatory roles of neutrophils in adaptive immunity. Cell Commun. Signal..

[45] Bergmann C.B., Beckmann N., Salyer C.E., Crisologo P.A., Nomellini V., Caldwell C.C. (2021). Lymphocyte Immunosuppression and Dysfunction Contributing to Persistent Inflammation, Immunosuppression, and Catabolism Syndrome (PICS). Shock.

[46] Öztürk Z.A., Yesil Y., Kuyumcu M.E., Bilici M., Öztürk N., Yeşil N.K., Özkaya M., Kısacık B., Kepekçi Y., Arıoğul S. (2013). Inverse relationship between neutrophil lymphocyte ratio (NLR) and bone mineral density (BMD) in elderly people. Arch. Gerontol. Geriatr..

[47] Zhou M., Li S., Pathak J.L. (2019). Pro-inflammatory Cytokines and Osteocytes. Curr. Osteoporos. Rep..

[48] Güler-Yüksel M., Hoes J.N., Bultink I.E.M., Lems W.F. (2018). Glucocorticoids, Inflammation and Bone. Calcif. Tissue Int..

[49] Hansson G.K., Robertson A.K.L., Söderberg-Nauclér C. (2006). Inflammation and atherosclerosis. Annu. Rev. Pathol..

[50] Forget P., Khalifa C., Defour J.P., Latinne D., Van Pel M.C., De Kock M. (2017). What is the normal value of the neutrophil-to-lymphocyte ratio?. BMC Res. Notes.

[51] Zahorec R. (2021). Neutrophil-to-lymphocyte ratio, past, present and future perspectives. Bratisl. Med. J..

